# Pulmonary Embolism Masquerading as High Altitude Pulmonary Edema at High Altitude

**DOI:** 10.1089/ham.2016.0008

**Published:** 2016-12-01

**Authors:** Prativa Pandey, Benu Lohani, Holly Murphy

**Affiliations:** ^1^ CIWEC Hospital, Kathmandu, Nepal.; ^2^ Tribhuvan University Teaching Hospital, Kathmandu, Nepal.

**Keywords:** extreme altitude, high altitude pulmonary edema, pulmonary embolism, thrombosis

## Abstract

Pandey, Prativa, Benu Lohani, and Holly Murphy. Pulmonary embolism masquerading as high altitude pulmonary edema at high altitude. *High Alt Med Biol*. 17:353–358, 2016.—Pulmonary embolism (PE) at high altitude is a rare entity that can masquerade as or occur in conjunction with high altitude pulmonary edema (HAPE) and can complicate the diagnosis and management. When HAPE cases do not improve rapidly with descent, other diagnoses, including PE, ought to be considered. From 2013 to 2015, we identified eight cases of PE among 303 patients with initial diagnosis of HAPE. Upon further evaluation, five had deep vein thrombosis (DVT). One woman had a contraceptive ring and seven patients had no known thrombotic risks. PE can coexist with or mimic HAPE and should be considered in patients presenting with shortness of breath from high altitude regardless of thrombotic risk.

## Introduction

Acute mountain sickness (AMS), high altitude cerebral edema (HACE), and high altitude pulmonary edema (HAPE) occur with a frequency of 40%–50%, 1%–2%, and 0.2%–6% at altitudes greater than 4000 m (Hackett et al., 1976; Maggiorini, 2010). Pulmonary embolism (PE) at high altitude is a rare occurrence that is described only in case reports in the high altitude medicine literature (Nakagawa et al., 1993; Shlim and Papenfus, 1995; Ashraf et al., 2006). While AMS, HAPE, and HACE resolve with the oxygen-richer air of lower altitudes following descent (Hackett and Roach, 2001; Murdoch, 2004; Imray et al., 2010; Luks et al., 2014), the diagnosis of PE requires definitive treatment and, if missed, may result in death. Severe pulmonary arterial thrombosis and pulmonary infarcts were noted in autopsies of four of seven trekkers in the Himalayas (Dickinson et al., 1983) with the remaining three noted to have cerebral edema; two with brain herniation. Before death, all patients had a history and/or findings suggestive of severe altitude illness and PE was not suspected despite one patient being anticoagulated after diagnosis of DVT. Reported death among trekkers from presumed altitude illness in Nepal ranges from 3.6 (Shlim and Gallie, 1992) to 7.7 per 100,000 trekkers (Leshem et al., 2008). It is possible that PE may have been responsible for some of these deaths.

Approximately 100,000 foreigners trek in Nepal annually (Nepal Tourism statistics 2014). From 2013 to 2015, over 1000 persons were seen at Canadian International Water and Energy Consultants (CIWEC) Clinic at 1400 m with altitude illnesses, including 217 with HACE and 303 with HAPE. Among 303 persons diagnosed with HAPE at initial presentation, 8 were shown to have PE by computed tomography pulmonary angiography (CTPA) and an equal number suspected of having PE had a negative CTPA. Pneumonia was suspected in 22 patients to co-exist with HAPE, whereas 3 persons were confirmed to have pneumonia as the primary diagnosis. We did not identify any patients in this cohort with congestive heart failure as the primary diagnosis though two persons with suspected HAPE had a final diagnosis of myocardial infarction based on ischemic changes on electrocardiogram (ECG) and diagnostic serum troponin elevations. Among all travelers seen at the CIWEC during the same period (24,000 for 3 years), PE was diagnosed in three cases without altitude exposure.

We describe and discuss in detail seven of the eight cases with PE diagnosed at our clinic. One of the eight patients declined to provide permission for reasons of privacy to be included in this case series description. An evaluation of these cases may provide clues to PE diagnosis in the altitude traveler and hopefully to raise awareness of PE and DVT at high altitude.

## Case No. 1

**FIG. 1.**
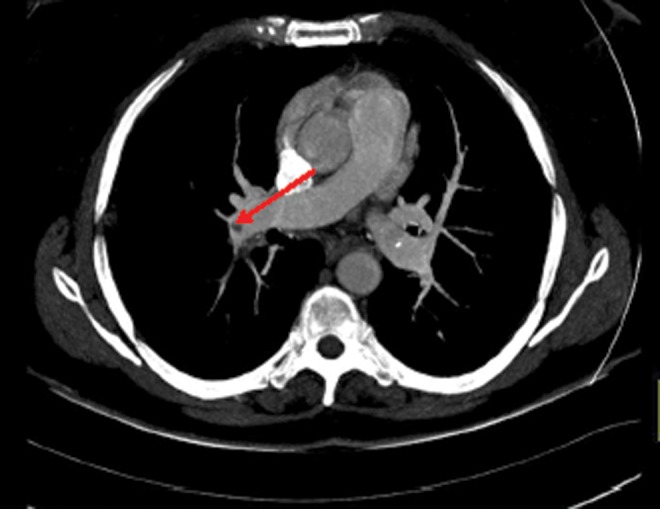
Patient 1: Axial image of computed tomography (CT) pulmonary angiogram with filling defect in right pulmonary artery (*arrow*) indicating thrombus.

A 64-year-old British female was evacuated by helicopter from the Everest region with symptoms of dyspnea and cough in October 2013. She reported a relatively slow rate of ascent to Dingboche (4400 m) when she developed severe headache and nausea. She noted chest pain and dyspnea with SpO_2_ 73% on ambient air. She descended slowly by foot to Tengboche (3800 m) and then to Namche (3440 m) without symptom improvement. She noted fatigue and dyspnea on exertion (DOE) with a few steps. She denied any significant past medical history and was not taking any medication. Upon arrival to the CIWEC, her SpO_2_ was 99% on ambient air though she remained dyspneic with a respiratory rate (RR) of 26 breaths per minute. Her lungs were clear to auscultation, and chest X-ray was unremarkable. She was thought to have residual HAPE and was administered O_2_. An echocardiogram showed mild mitral regurgitation (MR), mild tricuspid regurgitation (TR), and mild elevation of the systolic pulmonary artery pressure with no dilatation of right atrium (RA) or right ventricle (RV). In light of her persistent dyspnea, 24 hours after admission, a CTPA was done that documented thrombi in the right pulmonary artery and segmental branches of bilateral lower lobe pulmonary arteries (Fig. 1). Patchy consolidation was noted in the right middle lobe. Doppler studies showed normal deep veins in both lower limbs. She was treated with oxygen and anticoagulation and discharged with a therapeutic International Normalized Ratio (INR) after 6 days.

## Case No. 2

**FIG. 2.**
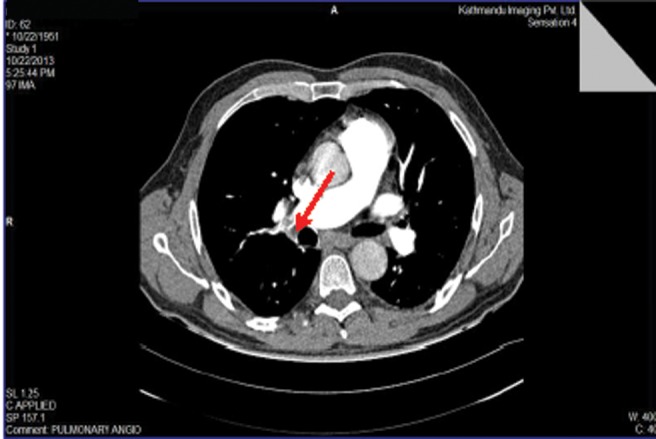
Patient 2: Axial image of CT pulmonary angiogram showing thrombi as filling defects in distal right pulmonary artery (*arrow*) with extension into right lower lobe branches.

A 62-year-old Swiss male climbing in the Manaslu region of Nepal in October 2013 developed pain and heaviness in the left calf 18 days into the trip at 6100 m. Four days later, he developed chest pains and dyspnea upon further ascent. He was evacuated by helicopter to CIWEC where he noted mild improvement in his respiratory symptoms. He had no significant past medical history and was not taking any medication. His SpO_2_ on ambient air was 89%; the lungs were clear and left leg swelling was noted. Electrocardiogram (ECG) showed nonspecific changes in the inferior leads and blood studies revealed normal troponin and creatinine kinase MB (CK-MB) isoenzyme concentrations. Polycythemia was noted with hematocrit of 54.8%; but all other laboratory studies were unremarkable. Doppler ultrasound confirmed DVT in the left popliteal and posterior tibial veins. His CTPA revealed thrombi in superior and inferior segments of right main pulmonary artery and bilateral lower lobe arteries (Fig. 2). Cardiac ultrasound showed mitral valve prolapse (MVP), mild MR, and trivial TR. The RV and RA were not dilated. There was no obvious thrombus visualized at the pulmonary artery (PA) bifurcation. He was treated with oxygen and anticoagulation and discharged after 5 days.

## Case No. 3

A 53-year-old American male was evacuated by helicopter from the Everest region in October 2013. He had a history of hypertension, hypercholesterolemia, and bilateral hip surgeries (2 and 6 years prior). He had kept to a relatively slow ascent rate after a flight to Lukla (2800 m) and with appropriate acclimatization before trekking to Khumjung and the Kongde Ridge (4250 m). He noted severe bilateral hip pain and cold from a drafty room, thus sleeping on an electric mattress intermittently for 16 hours. By morning, he noted dizziness and fell without sustaining any trauma due to a brief loss of consciousness. He also complained of DOE. He received oxygen and oral hydration prior to evacuation to Kathmandu by helicopter. Upon arrival, he was diaphoretic, tachycardic, and hypotensive (pulse 120/min, blood pressure [BP] 90/80 mm Hg, RR 24/min) with a SpO_2_ of 77% on ambient air, but felt some symptomatic improvement with the descent. His lungs were clear, and cardiac examination was normal. There was no leg swelling or tenderness.

**FIG. 3.**
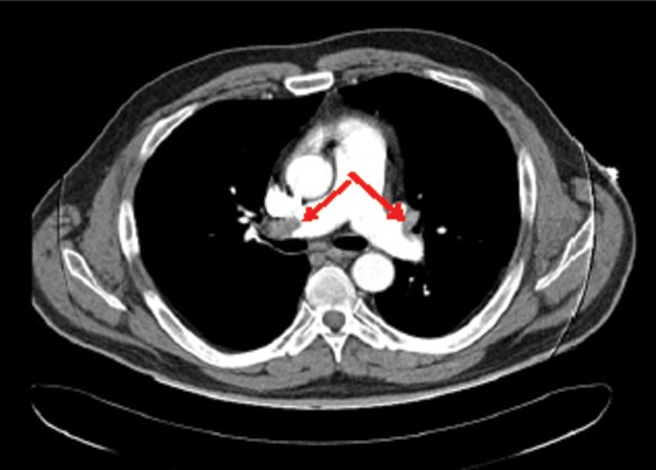
Patient 3: Axial image of CT pulmonary angiogram showing thrombi as filling defects in right main pulmonary artery (*right arrow*) extending into its branch and in distal left pulmonary artery (*left arrow*) with extension into its superior branch.

Laboratory evaluation revealed leukocytosis, renal insufficiency, and hyponatremia (White cell count 17,800/mm^3^, hematocrit 43.2%, BUN 20.4 mg/dL, serum creatinine 1.6 mg/dL, sodium 129 meq/L, potassium 3.4 meq/L, glucose 186 mg/dL). His chest radiograph was unremarkable. ECG showed T wave inversions in leads III, v3–5. Troponin and CK-MB concentrations were within normal limits. A CTPA done the same day confirmed thrombi in the right main pulmonary artery and segmental branches of left pulmonary artery (Fig. 3). He received oxygen 10 Liters by non-rebreather mask. An echocardiogram showed a mildly dilated RV with mildly elevated systolic PA pressure of 40 mmHg. A Doppler study revealed an acute DVT in the left popliteal vein. He was treated with oxygen and anticoagulation and was discharged after 5 days.

## Case No. 4

A 37-year-old German female presented in November 2013 with right leg pain for 3 days upon completion of a trek to the Everest region. She reported dyspnea and a dry cough at 4900 m. She was evacuated by horse to 2800 m with subsequent helicopter evacuation to Kathmandu. She was using a combined contraceptive vaginal ring (NuvaRing). She denied cigarette smoking. Presenting vital signs at the CIWEC were unremarkable (temperature 37.5°C, pulse 97/min, BP 110/80 mmHg, SpO_2_ 98% on ambient air) as were her lung and cardiac examinations. Edema and tenderness were noted in the right calf. She reported a slightly larger right calf at baseline due to a congenital large vascular nevus. Doppler ultrasound demonstrated an acute DVT extending along the mid one-third of the femoral vein proximally. Her ECG and echocardiogram were unremarkable. Her CTPA showed a non-occlusive hypo-dense thrombus in left lower lobe pulmonary artery. She was treated with oxygen, anticoagulation, and the contraceptive vaginal ring was removed.

## Case No. 5

A 70-year-old American male was trekking in the Langtang region in April 2013, 4 days after arriving in Nepal. He reported fatigue, cough, and dyspnea 4 days into the trek. He was evacuated to Kathmandu by helicopter from Magin Goth (3285 m). On arrival, he was dyspneic and afebrile with a RR 30/min and SpO_2_ 70% on ambient air, pulse 82/min, BP 130/80 mmHg. There were scattered crepitations in both lung bases. His white cell count was within normal limits. His echocardiogram showed mild TR, mildly elevated pulmonary artery pressure, and a mildly dilated RV. The chest X-ray showed non-homogeneous opacities in the mid and lower zones of both lungs with PA enlargement interpreted as consistent with HAPE. He received oxygen, amoxicillin–clavulanic acid, and azithromycin. He remained dyspneic requiring oxygen for more than 24 hours. A CTPA was done on the following day revealing thrombi in the segmental artery of right lower lobe. Venus Doppler Studies showed an acute DVT involving the left popliteal vein. He was anticoagulated and discharged after 8 days.

## Case No. 6

A previously healthy 71-year-old Austrian woman was evacuated from the Everest region from 4900 m on horseback to 4200 m due to severe respiratory distress and decreased responsiveness. Soon after arrival to the medical aid-post, she lost consciousness and pulses, and developed agonal breathing. She was treated with high flow oxygen, IV fluids, and dexamethasone with minimal improvement overnight, and remained somnolent with labored breathing at which point she was evacuated to Kathmandu. On arrival at the CIWEC, she was conscious, but dyspneic with RR at 32/min, pulse—100/min, temp—37.9°C, and SpO_2_ of 78%. Her lungs were clear, and cardiac examination was normal. The ECG showed T wave inversions in lead V1–3. Her CXR was normal and echocardiogram showed dilated RA/RV, mild TR, and mild elevation of the systolic pulmonary artery pressure. A CTPA confirmed thrombosis in the right and left pulmonary arteries and in their inferior and superior branches. Doppler ultrasound of both legs showed a normal deep venous system in both lower limbs with the exception of a superficial acute thrombosis in the right calf. She was treated with anticoagulation, oxygen, and was discharged to return to her home country.

## Case No. 7

A 49-year-old German male trekker was seen after helicopter evacuation from 4700 m 11 days into his trek. He developed dyspnea at an altitude of 4100 m, but was able to continue and cross the Teri La pass in Mustang at 5600 m. He developed increasing dyspnea, fatigue, cough, and intermittent chest pain despite descent to 4700 m and was helicopter evacuated to Kathmandu. At the CIWEC, he was tachypneic on arrival with other vital signs stable (RR 28–30/min, SpO_2_-96%, pulse 77/min, and temp of 37.1°C). There were bibasilar crepitations in the lungs, and the cardiac examination was normal. An ECG showed antero-lateral T wave inversions, the echocardiogram showed a dilated RA and RV, trace TR with mildly elevated systolic PA pressure. His CTPA showed multiple thrombi in the sub-segmental branches of the right upper and both lower lobe pulmonary arteries. Doppler ultrasound of both lower extremities demonstrated acute thrombosis of the deep veins of the left calf with extension into the posterior tibial veins. He improved with oxygen and anticoagulation.

## Summary

We present seven of the eight cases with preliminary diagnosis of HAPE who did not improve with descent and oxygen as expected and were diagnosed with PE following definitive imaging studies. The average age of the cases with PE was significantly older than that for HAPE in the same period at the CIWEC: 58 years (range 37–71 years) versus 45 years (range 20–74 years). Six of seven cases had been to altitudes >4000 m (Table 1). Five of seven patients developed symptoms after 10 or more days trekking at high altitude. Five of our PE cases were found to have DVT and one case had calf vein thrombosis on compression ultrasonography (Table 1).

**Table 1.** Table Showing Patient Details with Maximum Altitude Reached, Days at High Altitude, Initial Diagnosis, Time to Diagnosis of Pulmonary Embolism, Findings on Computed Tomography Pulmonary Angiography, and Doppler Ultrasound of Lower Extremities

*No.*	*Age/sex*	*Preexisting*	*Max alt (m)*	*Days at high alt (days)*	*Initial diagnosis*	*Time to CTPA (hrs)*	*CTPA*	*Doppler U/S lower extremities*
1	64/F	None	4400 m	10	HACE, HAPE	24	Thrombus R PA branches	(−) DVT
2	62/M	None	6100 m	20	HAPE DVT LLE	22	Thrombus in R main PA	DVT L popliteal
3	53/M	HTN, both hips replaced	4250 m	10	R/O PE HAPE	5	Thrombus R main, L, and R PA branches	DVT L popliteal
4	37/F	NuvaRing, vascular nevus	4900 m	14	HAPE, R/O DVT R/O PE	26	Thrombus in LLL PA	DVT R Femoral
5	70/M	None	3285 m	5	HAPE	24	Thrombus in RLL segmental PA	DVT L popliteal
6	71/F	None	4900 m	8	HAPE	8	Thrombus in R and L pulmonary arteries	Thrombus in muscular vein R calf
7	49/M	None	5600 m	11	HAPE	24	Thrombi in subsegmental branches of R upper and both lower lobes PA	DVT L popliteal

Alt, altitude; CTPA, computed tomography pulmonary angiography; DVT, deep vein thrombosis; F, female; HACE, high altitude cerebral edema; HAPE, high altitude pulmonary edema; M, male; Max, maximum; L, left; LLL, left lower lobe; PA, pulmonary artery; PE, pulmonary embolism; R, right; RLL, right lower lobe; R/O, rule out.

## Discussion

While HAPE is a common problem at high altitude in Nepal with rates of 1%–6% reported in the Everest region alone (Hackett et al., 1976; Basnyat et al., 1999), PE has been uncommonly reported among trekkers and climbers to Nepal (Dickinson et al., 1983; Shlim and Papenfus, 1995). Despite the current knowledge of altitude and pulmonary disease and scattered case reports, there is no clear association between PE risk and altitude. There are some data suggesting an increased risk among individuals with underlying coagulopathy (Schreijer et al., 2006; Khalil and Saeed, 2010). There are larger retrospective studies suggesting a prolonged stay at altitude alone may be a risk for PE (Anand et al., 2001; Khalil and Saeed, 2010; Rathi et al., 2012), but these data do not suggest greater risks for shorter stays, for example, of the trekker/mountaineer. Considering the lack of clear association between altitude and PE and the prevalence of HAPE as a cause of respiratory distress from altitude, there is often great difficulty in deciding which patients to evaluate for PE in this setting.

PE at altitude in our series is likely multifactorial—including modifiable and unanticipated factors—and may present in altitude travelers without prior risk factors. Despite the lack of evidence that short-term travel to altitude increases PE risk, there are aspects of altitude excursions that increase blood viscosity, for example, dehydration causing hemoconcentration, and polycythemia, in addition to the compensatory rise in hematocrit with acclimatization, which are likely to increase PE risk. In our series, one patient was noted to have polycythemia. Though a prolonged journey prior to trekking poses an obvious risk, most of the patients presented here developed symptoms late in their trek suggesting that this was not a risk factor. Only one elderly traveler who developed respiratory symptoms at lower altitude (3200 m) had trekked within 7 days of an international flight, suggesting that short time interval after his flight may have been a risk in this case. Furthermore, we have noted 3 fold more PE cases occuring with altitude than at lower altitude in the last 3 years. The majority of these cases at altitude became ill above 4000 m, which may support prior work suggesting a role for increasing altitude in thrombosis (Gupta and Ashraf, 2012). Still, none of these factors distinguish PE from HAPE.

The average age of our PE cases was significantly higher than that for HAPE patients. The risk of phlebitis and PE has been shown to be higher for age >60 versus younger travelers (Gautret et al., 2012). However, selecting patients for evaluation simply based on age would still result in missed PE diagnoses, considering three of seven patients in this series were less than 55. In this series, only one patient had a known thrombotic risk–a vaginal contraceptive ring, which has similar prothrombotic risk as combined oral contraceptives (Kolacki and Rocco, 2012; Nguyen and Jensen, 2014; Kenmuir et al., 2015; Paresi et al., 2015). Based on this and prior reports (Shlim and Papenfus, 1995; Schreijer et al., 2006; Khalil and Saeed, 2010), we conclude that any thrombotic risk factor should warrant a PE work-up for hypoxemia and any respiratory symptoms or findings at high altitude.

Initial clinical, laboratory, and radiographic presentation provided clues to the PE diagnosis in the 2 cases with DVT symptoms (Cases 2 and 4) and for 1 patient (Case 3) who was hypotensive and hypoxemic on arrival with a negative cardiac workup and normal chest X-ray. Another four were persistently dyspneic after descent and therefore underwent CTPA. Other data-including ECG, CXR, and echocardiogram findings among these patients did not vary significantly from patients with “pure” HAPE. The clinical assessment tools e.g. the Wells criteria that take into account recent surgery, immobility, and presence of malignancy were not useful in identifying risk among these patients (Wells et al., 2000). We do not screen patients with HAPE with D-dimer testing despite the high sensitivity (Righini et al., 2014; Woller et al., 2014) due to the low specificity that can lead to unnecessary imaging studies, which may also introduce further hazard to the hypoxic patient (Rathi et al., 2012; Bokobza et al., 2014). Furthermore, there is evidence that D-dimer levels increase with altitude in the absence of overt thrombosis (Pichler Hefti et al., 2010). We are not aware that we missed any cases of significant PE with this approach. We conclude that a thorough clinical evaluation and high index of suspicion if the response to HAPE treatment is poor remains our most sensitive diagnostic strategy for considering PE in this setting.

Theoretically, systemic endothelial dysfunction-recently shown to occur in HAPE cases with finding of raised endothelin-1 (ET-1) levels (Droma et al., 1996; Sartori et al. 1999; Berger et al. 2005; Charu et al., 2006; Barker et al., 2016), may also be elevated with PE. Efforts to identify rapid tests for HAPE will need to differentiate between these potentially overlapping and confounding diagnoses.

We conclude that PE, a diagnosis with significant mortality, may be missed in the traveler at high altitude due to similar presenting symptoms as occur with HAPE. Based on our experience, including the cases presented here, we propose obtaining a CPTA or other imaging such as a nuclide ventilation-perfusion scan when patients presenting with symptoms suggestive of HAPE who do not improve or get worse with descent within 24 hours. Moreover, diagnostic imaging should be considered if a DVT is suspected when symptoms develop during descent, and when significant chest pain is present and other etiologies such as myocardial infarction are ruled out and lastly for patients with any known or perceived thrombotic risk. Due to the high fatality of PE and the overlap in clinical presentation with HAPE, a more aggressive approach to PE diagnosis is warranted in high altitude travelers with respiratory symptoms.
